# The Value of Formaldehyde in Septic Root Canals

**Published:** 1914-01-15

**Authors:** Frederick W. Frahm

**Affiliations:** Denver, Colorado


### THE VALUE OF FORMALDEHYDE IN SEPTIC ROOT CANALS.

BY FREDERICK W. FRAHM, PH.G., D.D.S., DENVER, COLORADO.

Since it seems that everything new from a nation down to a hairpin has its ups and downs, its times of favor and shadows, the same can be said of the formaldehyde preparations, and at this time these seem to be in the shadows of disfavor. I think I should say seeming disfavor, because I fear that as clinicians and therapeutists we sometimes jump to conclusions without giving the drug which we condemn a fair trial.

The use of formaldehyde seems to have been based a good deal on the theory, that, "If a little is good, more is better." When a fraction of a grain would have been sufficient, three or four times that amount has been used.

There was a time when mercurous chloride was used in large doses, even up to ten grains per dose. We have all heard of the effects of this dosing, if we may not have been permitted to see it. May I ask, did the medical profession discard this very valuable drug? No, I dare say not, for we all know what place it holds in the pharmacopea and the materia-medica of to-day, only it is now given with excellent results in from one-tenth to one-fourth grain doses. And I do not know of another drug so valuable, nor of one that can take its place.

The same may be said of formaldehyde. It has at this time found disfavor at the hands of some, not, I think, on account of its uses, but because of its misuse.

I am not going to formulate a fine theory as to the chemistry of what occurs or may occur in a putrescent tooth canal or pulp chamber. Just to call your attention to the fact that the products of decomposition of animal tissue bring into being many compounds, among which we find sulfuretted hydrogen, ammonia and its compounds, carbon dioxide, water and some solids. To this may be added some of the decaying foodstuffs.

Formaldehyde gas is a hydro-carbon with a formula of H.CHO, capable of being absorbed by water and other compounds, forming new compounds, which may be acted upon by other agents forming condensation products, such as, glutol with proteids; dextroform with carbohydrates; tannaform with urea and products containing tannin; urotropia with ammonia; and so on. I might mention many more. These condensation products are non-irritating and very valuable antiseptics, many of which, and especially glutol, tannopin, tannoform, helmitol, urotropin, and citerin are given internally for various systemic troubles. Since this chemical action takes place, and the elements necessary for forming these condensation products are found in the average putrescent tooth canal, formaldehyde in some form is indicated.

I do not agree with the methods advocated by some for its use, nor do I approve the technic followed out in the treatment, though favorable results have been obtained. I prefer to use paraform (triformal) or solidified formaldehyde. By its use I can know just the amount that is placed in the tooth and where it is being placed, and can look for and expect certain actions to take place with certain results.

I agree with Dr. Grove, of St. Paul, Minn., that much injury has been done by this powerful agent. I have seen much of the soft tissue between the teeth injured or destroyed by its careless use, for it must not be forgotten that we are dealing with a powerful escharotic. Also, some apical troubles have resulted from its use in root canals. But I sincerely believe these results have been brought about by its abuse and through carelessness.

I do not believe that it is necessary to add any essential oils or anodynes to it. The drug itself is an anodyne and does not depend upon any other drug or agent as an aid to its action. The weeping at the apex of the root or the eczema attendant upon its action on the mucous membranes need not take place unless the technic has been faulty.

I did not start out to write a paper on septic root canals, nor do I intend to give an extended treatise on the pharmacology, materia-medica and therapeutics of formaldehyde. I simply want to say a word in its defense and praise, feeling certain that it is worthy of all the praise that I can give.

As stated before, I prefer the solidified formaldehyde. It is easy to use. You can cut out as much as you desire and place it where you wish with a degree of certainty that the other preparations do not permit; neither do you run the risk of having it leak from the cavity.

### TECHNIC FOR USING FORMALDEHYDE.

After opening the pulp chamber (if it is not already open) with a spoon excavator, clear it of all decayed matter and flush well with warm water. Now adjust the rubber dam on two or more teeth, if it is an approximal cavity, and dry the cavity and pulp chamber well, removing the decay from the gingival seat and walls to admit of perfect sealing with cement. Do not make any attempt to cleanse the canals, nor even enter them with an instrument; simply place a small piece of the drug, about as much as a pin head for the bicuspids and the anterior teeth and twice that amount for the molars, on the sub-pulpal walls; upon this place a small piece of cotton soaked in cresol. I use cresol, not for any effect that it may possess as an anodyne, but simply as an aid to subsequent treatment. Then seal with cement, making sure there is no leak, especially at the gingival seat.

If this work has been carefully done, no matter how foul or sore the tooth was at the first sitting, when the patient returns several days hence he will tell you, "Doctor, within half an hour after I left the office the other day the tooth was comfortable, and has felt fine ever since."

I have used this drug and technic since 1903, and can say that the results obtained have been gratifying to me. I do not think I could record more than four or five per cent. of failures, and these were due to necrosis or absorption of the roots. I have in many cases called the X-ray to my assistance to determine the condition at the apical area, also to verify my work on the root canal fillings. In no case was there any destruction of tissue in the apical area, nor did time develop any untoward symtoms. I am fully aware of the fact that dentists do not always get an opportunity to see their failures. I have been permitted to see enough of my cases to warrant the above statement.

At the second sitting apply the dam and freely open the pulp chamber, if it has not been done before. To your great surprise all the malodor will be absent and you can only detect the cresol. The work of the formaldehyde has been accomplished; you do not need it again for this tooth. Now thoroughly cleanse the canals with the proper instruments, wipe out with alcohol and dry with hot air. Dress the canals and pulp chamber with cresol or campho-phenique, seal with cement and dismiss the patient till the next sitting, when the tooth and canals may be filled, with an assurance of success. In two exceptional cases I have given the tooth three treatments.

REMEMBER.

For the first sitting. 1. Do not try to cleanse the canals either chemically or mechanically. 2. Remove with the proper excavators the decay at the gingival seat and other margins. 3. Do not place any of the drug up into the canals; place it over the openings of them, or in the pulp chamber. 4. Seal the cavity as perfectly as is possible with cement, being careful to avoid pressure. If these four instructions are ignored you are liable to have a lame tooth.—*Items of Interest*, Dec. 1913.

